# The slings and arrows of comparative linguistics

**DOI:** 10.1371/journal.pbio.3000019

**Published:** 2018-09-24

**Authors:** Johan J. Bolhuis, Gabriel J. L. Beckers, Marinus A. C. Huybregts, Robert C. Berwick, Martin B. H. Everaert

**Affiliations:** 1 Cognitive Neurobiology and Helmholtz Institute, Department of Psychology, Utrecht University, Utrecht, the Netherlands; 2 Department of Zoology and St. Catharine’s College, University of Cambridge, Cambridge, United Kingdom; 3 Utrecht Institute of Linguistics, Utrecht University, Utrecht, the Netherlands; 4 Department of Electrical Engineering and Computer Science, Massachusetts Institute of Technology, Cambridge, Massachusetts, United States of America

## Abstract

In this Formal Comment the authors respond to objections to their previous Essay, reiterating that comparative linguistics is not an easy undertaking.

There are intriguing avian parallels to human speech acquisition absent in our closest relatives, the great apes [1–3]. Although unbounded hierarchical syntactic structure is thought to be unique to human language, there is no a priori reason why the computational mechanisms used to build such structure could not have evolved in other animal species [4]. So far, however, there is no evidence to support such a claim [2–5]. Only extensive and thorough comparative work can provide insights into which features of the language faculty are uniquely human and which ones are shared with other animals. We therefore welcome the contributions of Suzuki and colleagues [6,7] and Engesser and colleagues [8], as well as their responses to our essay [9,10]. Our critical evaluations of their claims on compositionality in birds [5] are not in any way linked to the value of a comparative biological approach. Nor do we think that such an approach would be rendered obsolete, even if a single computational operation of recursive combination turns out to underlie all human syntax, a position held by Townsend and colleagues [10]. In fact, these authors go to great lengths arguing against something that we are actually not discussing—whether or not the Minimalist Program is an appropriate theoretical linguistics framework. Such questions are better addressed elsewhere.

We are pleased that the authors agree with us on what constitutes the basic property of the language faculty: unboundedness (“generat[ing] innumerable expressions from a finite number of vocal elements and meanings” [9]) as well as its unique structural features (“creat[ing] exceedingly complex dependencies” [10]). Suzuki and colleagues [9] also agree with us on distinguishing a Faculty of Language in the Narrow sense (FLN) (“Human language is no doubt unique in the complexity of its expressions” [10]) from a Faculty of Language in a Broad sense (FLB) (“providing insights into possible evolutionary drivers of these faculties of language [in a broad sense]” [9]). Comparative biology should provide insight into what aspects belong to FLN and what to FLB [11].

Although hierarchical structure is abundantly grounded in empirical linguistic and psycholinguistic facts and remains the consensus view in contemporary formal linguistic theories [4,5], Townsend and colleagues [10] suggest that language must be viewed as consisting of a number of components on an equal level (and not in a subset relation as advocated in [11]). In this way we must understand “rudimentary compositionality” as one of these components of language (shared by some species of birds and humans), on which full recursive compositionality is built. We certainly do not disagree with the observation that languages possess “an extensive layer” of language use that might be described as nonproductive or even nonhierarchical. However, we find that Townsend and colleagues [10] draw a false and misleading distinction between “simple/non-hierarchical vs. complex/ hierarchical.” It is not clear that nonhierarchical mechanisms are simpler than hierarchical ones. The important question is whether “complex” structure is generated by different rule systems than “simple” structure. The burden of proof is on those who posit an additional mechanism for “simple syntax” besides a single, simple computational rule that in addition to “complex syntax” also generates “simple syntax.” It is exactly to avoid such confusions that one should be precise when talking about what “language” is. We must distinguish language as a human cognitive trait (a mind-internal biological system) from its specific manifestation (verbal behavior), whether “simple” or “complex”.

Central in the debate is whether these authors [6–8] have shown that the investigated bird species exhibit behavior that might indicate functional “compositionality.” We have explained that what the authors call “compositional syntax” has nothing to do with “compositionality” as conventionally defined [12], and Townsend and colleagues [10] do not question this point. We are pleased to see that Suzuki and colleagues [9] now provide an explicit [12] definition of compositionality: “[…] where the meaning depends on both the meaning of its parts and the way in which they are combined.” But it is still not clear what is meant here, given that Hurford [13] also discusses an alternative formulation: “Lexical syntax, or lexicoding, as Marler calls it, is the kind of putting things together where the elements mean something, and the whole assembly means something which is a reflection of the meanings of the parts. This is **compositionality**. Complex meanings are expressed by putting together smaller meaningful units.” ([13], p. 335; bold emphasis in the original). We simply want to point out that both of Hurford’s descriptions leave open the possibility that “structure” is not part and parcel of the notion of compositionality. This mistaken notion of compositionality was the theoretical foundation of the original papers [6–8], and we discussed it in some detail in our essay [5]. Unfortunately, Suzuki and colleagues [9] still do not provide any evidence for genuine compositionality in these bird vocalizations. They now acknowledge “[…] that changing the order in which the calls are combined does not produce an alternative compound message, but, rather, a sequence with unclear or ambiguous meaning.” [9]. Clearly, XY being meaningful and YX meaningless is what we already pointed out ([5], Box 2) and need have nothing to do with compositionality. In natural language, XY and YX will both have meaning. One of them may be infelicitous or communicatively inappropriate but would have meaning, distinguishing it from what is pointed out by Suzuki and colleagues [9]. They muddy the waters here considerably by adding mistaken assumptions. First, “ambiguity” arises pervasively in language both at the level of words (lexical ambiguity) and phrases (structural ambiguity) and doesn't care about two-word (“visiting relatives”) or many-word constructions (our paper). Second, linguists do not take “written language” as their object of study. Third, “in many cases word order matters.” Repeating ourselves, word order does not, for instance, matter for the semantics of simple modification [10] in French examples like “arbre vert/*vert arbre” (green tree) and “vieux arbre/*arbre vieux” (old tree). The adjective–noun word order difference counts in French but is not relevant for the semantic buildup of the phrase.

Suzuki and colleagues [9] point out that the definition of “compositionality” does not refer to hierarchical structure and free productivity. But the notion of compositionality, as given by us, makes little sense if one does not assume both. The linear structure of spoken, signed, or written language results simply from the physical limitations of externalization; it is not central to what syntactic structure is about [14]. Without productivity, a phrase and the meaning associated with it might just as well be listed, making, by the way, “proto forms of compositional syntax” superfluous as an “intermediate compositionality” step [10]. It is precisely the power of productivity and the empirical result that phrases are hierarchically structured that makes assigning meaning to structure a lot less complex.

## Abbreviations

FLN: Faculty of Language in the Narrow sense
FLB: Faculty of Language in a Broad sense
